# A New Volumetric Fusion Strategy with Adaptive Weight Field for RGB-D Reconstruction

**DOI:** 10.3390/s20154330

**Published:** 2020-08-03

**Authors:** Xinqi Liu, Jituo Li, Guodong Lu

**Affiliations:** 1 Institue of Design Engineering, School of Mechanical Engineering, Zhejiang University, Hangzhou 310027, China; liuxinqi@zju.edu.cn (X.L.); lugd@zju.edu.cn (G.L.); 2 Robtics Institue, Zhejiang University, Yuyao 315499, China

**Keywords:** volumetric fusion, RGB-D reconstruction, texture reconstruction, data evaluation

## Abstract

High-quality 3D reconstruction results are very important in many application fields. However, current texture generation methods based on point sampling and fusion often produce blur. To solve this problem, we propose a new volumetric fusion strategy which can be embedded in the current online and offline reconstruction framework as a basic module to achieve excellent geometry and texture effects. The improvement comes from two aspects. Firstly, we establish an adaptive weight field to evaluate and adjust the reliability of data from RGB-D images by using a probabilistic and heuristic method. By using this adaptive weight field to guide the voxel fusion process, we can effectively preserve the local texture structure of the mesh, avoid wrong texture problems and suppress the influence of outlier noise on the geometric surface. Secondly, we use a new texture fusion strategy that combines replacement, integration, and fixedness operations to fuse and update voxel texture to reduce blur. Experimental results demonstrate that compared with the classical KinectFusion, our approach can significantly improve the accuracy in geometry and texture clarity, and can achieve equivalent texture reconstruction effects in real-time as the offline reconstruction methods such as intrinsic3d, even better in relief scenes.

## 1. Introduction

With the increasing importance of three-dimensional reconstruction technology in the fields of automatic driving, virtual reality, robot positioning, and navigation, the reconstruction of high-quality 3D scenes using low-cost consumer-grade RGB-D sensors has become a hot issue and has been widely studied.

**Figure 1:**
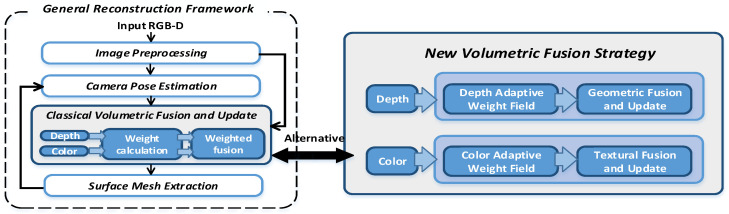
The comparison in the overall framework between the classical voxel fusion method and our approach. On the left side of the figure is a general reconstruction framework using classical fusion methods. On the right of the figure is our new fusion strategy that can be used as a basic module and directly applied to various volumetric construction frameworks.

The general framework for reconstructing static scenes consists of five parts: input RGB-D data stream, image preprocessing, camera pose estimation, volumetric fusion and update, and surface mesh extraction, as shown in the left image in Figure 1, among which the volumetric fusion step plays an especially important role. Classical volumetric fusion strategy usually uses point-based sampling and weighted average to calculate surface geometry and texture information. For the geometry, this method works well because it is based on the assumption that the depth data noise obeys Gaussian distributions with zero means. Therefore, through repeatedly sampling and integrating, the geometric noise can be effectively reduced to get a smooth reconstruction surface. However, for textures, if the weighted average fusion strategy is directly applied to texture fusion, it will lead to obvious blurring problems. The main reason is that due to the existence of camera pose estimation errors, the pixel information sampled from different RGB-D frames cannot match perfectly. The depth difference between these mismatched pixels is usually small, which will only affect the geometric fusion result slightly, but its color difference can be obvious, especially for texture-rich scenes where the colors on neighborhoods usually change sharply. By averaging the colors on such pixels to get the texture will inevitably lead to texture blur.

Generally speaking, classical voxel fusion methods have two shortcomings: (1) the weighted averaging methods are not suitable for texture fusion since they tend to destroy the texture structure and cause color blur. (2) Current data quality evaluation models are mostly concerned with depth information while ignoring the RGB information that however is crucial to the visual appearance.

Current research methods on improving the texture quality of the reconstruction results can be divided into two types.

The first type is attempts to adopt high-quality data for fusion. View-dependent [1,2] or various uncertainties based [3] probability models have been established to evaluate the confidence of the depth data to guide voxel fusion. But these kinds of methods do not consider the error that the color images may introduce. On the other hand, some algorithms have been designed to recover clear and high-quality data from blurred RGB frames [4] to reduce input errors. Although this kind of method considers the clarity of color images, it still cannot solve the problem of wrong textures in the edge area of the scene. More importantly, even though these methods can obtain relatively high-quality data, they usually use classical point-based sampling and weighted average methods without distinguishing texture fusion from geometry fusion, which inevitably leads to texture blur.

The second type is to use a joint optimization method to refine and correct the texture of low-quality reconstruction results that are obtained by using classic reconstruction frameworks such as KinectFusion. For example, a joint optimization method combines texture and geometry with the camera poses and lighting illuminations are designed to generate high clarity texture results [5]. Such a method requires a good initial reconstruction result and has a heavy computing burden and is hard to run in real-time.

To effectively reduce texture blur and generate a high-quality texture, we design a new volumetric fusion strategy from two aspects: (1) evaluate and select high-quality data for fusion and (2) design a texture fusion method that can preserve texture structure. As shown in the right side of Figure 1, we use a probabilistic and heuristic method to build an adaptive weight field, which can effectively evaluate and adjust the reliability of RGB-D data to guide voxel fusion by considering the consistency of texture structure features, the risk of wrong texture and the outlier noise of depth map. Then, we introduce a new texture fusion strategy that combines replacement, integration, and fixedness operations to generate texture results with accurate texture structure, thus texture blur can be effectively alleviated.

**Figure 2:**
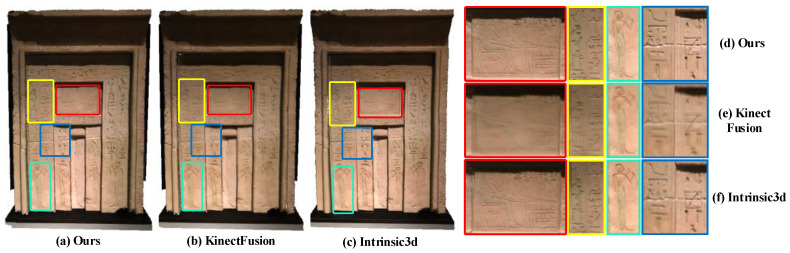
Texture reconstruction results of the Gate dataset. Our method (**a**,**d**) can achieve better results than the classical weighted average fusion method [6] (**b**,**e**) and the off-line Intrinsic3d reconstruction method [5] (**c**,**f**) in texture clarity.

Extensive qualitative and quantitative experiments and comparative analysis show that our method can remarkably improve the accuracy of geometric and texture reconstruction compared with classical weighted average fusion methods, and can achieve no less or even better results than the state-of-art off-line reconstruction methods. As demonstrated in Figure 2.

Overall, our technical contributes are as follows:

1. A new texture fusion strategy combining replacement, integration, and fixedness operations is proposed. It can effectively preserve texture structure information to generate accurate texture results with high clarity.
2. An adaptive weight field is established effectively by using the combination of probability and heuristics, which can evaluate and adjust the reliability of RGB-D data. This adaptive weight together with a refined camera pose estimation method are used to guide the voxel fusion process to avoid texture blur, wrong texture, and rough surface.
3. Our method can be easily embedded in the current online and offline reconstruction framework as a basic module, and can effectively improve the accuracy of reconstruction results.

The rest of the paper is organized as follows. After reviewing the related works in Section 2, the overall method is overviewed in Section 3. The strategies of geometry fusion and texture fusion are proposed in Section 4. To support high-quality fusion, the data reliability of each RGB-D frame is evaluated as an adaptive weight field in Section 5. A refined camera pose estimation method is established to reduce the mismatch between adjacent RGB frames in Section 6. Experimental results are presented in Section 7. The whole paper is concluded in Section 8.

## 2. Related Work

### 2.1. Weighted Averaging Volumetric Fusion

Weighted averaging volumetric fusion has become a basic component of many online [6,7,8,9] and offline [10,11,12] RGB-D reconstruction frameworks due to its easy calculation and high parallelism. KinectFusion [6] is the first real-time volumetric fusion method based on truncated signed distance function [13], which has achieved impressive results, making the volumetric fusion method widely recognized and studied. Subsequently, combining this fusion method with a deformation graph [14], different methods such as DynamicFusion [15], Fusion4D [16], KillingFusion [7], and SobolevFusion [17] realize the real-time reconstruction of the dynamic scene, which greatly expands the application scope of this kind of method. Doublefusion [18] effectively improves the robustness of dynamic reconstruction by introducing SMPL [19] body model as a priori to constrain the motion range. To improve the accuracy of reconstruction results, Zollhöfer et al. [20] proposed a fusion optimization strategy by changing the level of voxel size from coarse to fine to preserve geometric details. To solve the memory consumption problem of volumetric fusion in large scenarios, Niesner et al. [21] proposed a voxel hashing method to store voxel by using hash functions and fuse voxels information when necessary, which effectively reduces the required memory. Li et al. [22] introduced the octree voxel structure to represent surface details at different scales and used point and line features to improve the pose estimation results. The above works are all based on weighted averaging fusion. They can be well used in geometry reconstruction, but are not suitable for texture reconstruction because it will destroy the texture structure of the scene and tend to generate blur texture results.

### 2.2. Data Evaluation Model of RGB-D Images

Data evaluation model of RGB-D Images has been widely studied to effectively reduce the noise on the RGB-D images acquired from commercial times-of-flight (ToF) sensors such as the Microsoft Kinect v2, Intel RealSense, or Google Tango. The noise of this kind of sensor comes from many factors, such as distances, viewpoint angles, scene materials, ambient lighting, normal vectors of scene surfaces, etc. Different models have been developed to describe the noise distribution [23,24,25]. For example, to evaluate the noise distribution of depth images output by a ToF camera, Reynolds et al. [26] used the random forest algorithm to establish a confidence map of depth images that is trained based on the data collected by a lidar. Jordt et.al [27] used a polynomial interpolation Gaussian distribution based on the gradient to describe the noise of RGB-D images. Cao et al. [3] established a Gaussian probability model considering the measurement uncertainty and surface sampling uncertainty to describe the impact of noise. Jenke et al. [28] established a probabilistic mixture model consisted of truncated Gaussian distribution and uniform distribution to describe the noise using smooth and dense priors. Lefloch [29] used Mahalanobis distance to measure the anisotropic noise in the depth map, which effectively improved the accuracy of geometric results. The above noise models mainly suppress the noise in the depth map, so they improve the geometric accuracy well. However, they can not directly be used for color images. For the color images, although some clarity evaluation methods [30] have been proposed, the existing data evaluation model cannot effectively avoid the occurrence of texture blur and wrong texture problems.

### 2.3. Texture Accuracy Improvement Method

Texture reconstruction based point sampling and fusion are mainly used in real-time RGB-D reconstruction, and its accuracy improvement methods are mainly divided into two categories. The first selects high-reliability data for volumetric fusion [31], the second generates high-quality models by correcting low-quality construction results through optimization methods [32,33,34]. For the former, Klose et al. [4] proposed a scene-space-based sampling and filtering method, which can obtain a clearer color image by fixing blurred raw data from video streams, then improve the reconstructed texture accuracy. Cao et al. [3] proposed a surfel-based cloud fusion strategy using uncertainty modeling, and selected high confidence point data for fusion. For the latter, Maier et al. [5] proposed a joint optimization algorithm that combines geometry, texture, camera postures, camera model, and ambient illumination to obtain high-quality results by correcting a rough model reconstructed by KinectFusion. Furthermore, Guo et al. [35] proposed an optimization method that combines geometry, texture, illumination, and non-rigid deformation fields to achieve dynamic scene reconstruction with a good texture effect. However, on the whole, these data-based approaches rely heavily on data evaluation models and will inevitably produce blur problems. These optimization-based methods require good initial models, high computational cost, and complicated programming design, which makes them low portable and unsuitable for real-time applications.

## 3. Framework Overview

**Figure 3:**
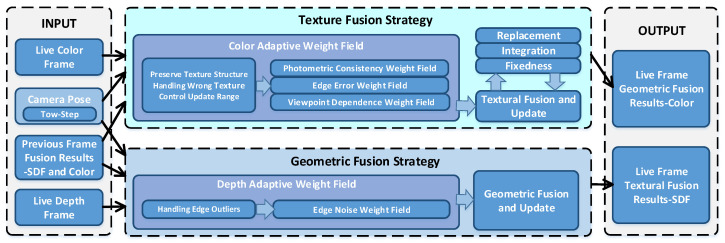
The specific pipelines for our new volumetric fusion strategy. We use the same input and output as the classical volumetric fusion method, and use different fusion strategies for geometric fusion and texture fusion respectively. For texture fusion strategy, we first establish an adaptive weight field to evaluate the confidence of the data and then use different operations to update the texture.

In our pipeline, geometry reconstruction and texture reconstruction are separated, and different update strategies are used to effectively save the texture structure of the reconstructed mesh to reduce the problem of texture blur. Consequently, high clarity texture can be generated, as illustrated in Figure 3. In this figure, there are three modules, the left and the right are the input and output that are consistent with the classical fusion method. In the input module, we propose a joint two-step camera pose optimization method that is helpful to improve the accuracy of reconstruction results. The middle is our new volumetric fusion strategy which consists of two parts:

(1) The adaptive weight fields describe the reliability of depth data and RGB data, respectively, to clearly distinguish the high-reliability data from input data by considering the consistency of texture structure features, the risk of wrong texture, and the outlier noise of depth map.
(2) In the fusion update part, a new texture fusion strategy combining replacement, integration, and fixedness is proposed to effectively reduce texture blur by considering the characteristics of texture structure.

When inputting the real-time frame, camera pose, and the result of previous fusion, our method firstly calculates the adaptive weight field of depth data and RGB data, respectively. Then, we fuse and update the geometry and texture of voxels with the calculated adaptive weight field.

The main purpose of building the adaptive weight field is to solve the problem of texture blur, wrong texture, and the influence of depth outlier noise on the geometric surface by evaluating and adjusting the reliability of RGB-D data. By establishing the photometric consistency weight field, we can successfully evaluate the local consistency of texture features between frames in the image domain, to provide highly reliable texture data for texture fusion and maintain the accurate texture structure of the global mesh. The edge error weight field is established to evaluate the high-risk areas where error textures are prone to occur. By adjusting the reliability of these areas, the problem of wrong textures can be effectively avoided. The edge noise weight field describes the characteristics of outlier noise in the edge region of the depth map. By adjusting its reliability in this weight field, the influence of these noises on the geometric surface can be effectively suppressed. By establishing these weight fields, we can provide high-reliability data for the voxel updating process and achieve accurate geometry and texture results.

In the voxel update process, we fusing the depth data and RGB data with different strategies. For the depth data fusion, since the weighted averaging method is suitable for geometry fusion, we use a similar method as the classical fusion approach. But we add a truncation term of low-confidence weights (CW) to discard the low-quality data from fusion, thus more precise surface reconstruction results can be obtained. For texture fusion, we propose a new volumetric fusion strategy that combines data operations of replacement, integration, and fixedness to fuse and update voxel textures. Its main idea is to introduce replacement and fixedness operations to retain texture structure information. The principal purpose of preserving integration operation is to make the structural texture edge change as smooth as possible and avoid the seam problem. By judging the variation of the confidence of the adaptive weight field estimation along with frames, we update the texture of voxel accordingly with the above three operations to preserve texture structure and reduce texture blur.

As camera pose is crucial to generate high clarity texture, we accurately estimate it with a two-steps optimization. We firstly use frame to a frame iterative closest points (ICP) method to estimate the initial camera pose, and based on this initial pose to establish the adaptive weight field which flags the reliability of RGB-D data; Secondly, we refine the camera pose by adopting the high-reliability texture data for ICP. The refined camera pose is reversely used to improve the quality of reconstructed texture.

## 4. New Volumetric Geometry and Texture Fusion Strategy

### 4.1. Geometry Fusion Strategy

We follow the classical fusion method to update the geometry with the alteration that the truncation function $\varphi_{d}\left(\cdot \right)$ and the fusion weight is calculated from the adaptive weight field rather than simply setting it to 1 or setting by viewpoint-dependence. This method ensures that the image information can be treated differently based on its reliability in the fusion process especially for the processing of outlier noise. So the geometric fusion strategy can be written as

(1) $$D_{i+1}\left(x\right)=\frac{W_{i}\left(x\right)D_{i}\left(x\right)+\varphi_{d}\left(w_{i+1}^{DAWF}\left(x\right)\right)d_{i+1}\left(x\right)}{W_{i}\left(x\right)+\varphi_{d}\left(w_{i+1}^{DAWF}\left(x\right)\right)}$$

(2) $$W_{i+1}\left(x\right)=W_{i}\left(x\right)+w_{i+1}^{DAWF}\left(x\right)$$

where $w_{i+1}^{DAWF}\left(x\right)$ is the weight value of voxel corresponding to the depth adaptation weight field of the *current* frame. $d_{i+1}\left(x\right)$ is the signed distance function value on the voxel center calculated in the current frames. $D_{i}\left(x\right)$ and $D_{i+1}\left(x\right)$ are the signed distance function values on the voxel center calculated from the previous *ith* frames and the current frame. $W_{i}\left(x\right)$ and $W_{i+1}\left(x\right)$ are the fusion CW calculated by previous *ith* frames and the current frame. $\varphi_{d}\left(\cdot \right)$ is a low CW truncation function that discards information with low confidence.

(3) $$\varphi_{d}\left(x\right)=\{\begin{array}{cc} x & x>0.2\\ 0 & else \end{array}$$

### 4.2. Texture Fusion Strategy

To effectively preserve the texture structure of the scene and reduce texture blur, we propose a new texture fusion strategy combining replacement, integration, and fixedness operations to obtain a clearer texture.

Our core ideas are as follows: when the reliability of the current frame sampling information is much higher than that of the previous fusion results, the previous result will be replaced by the data of the current frame. On the contrary, the fixedness operation is used to retain the high-quality fusion results of the previous frame. When the current frame is similar to the previous result in reliability, the data in the current frame will be integrated into the previous result.

**Figure 4:**
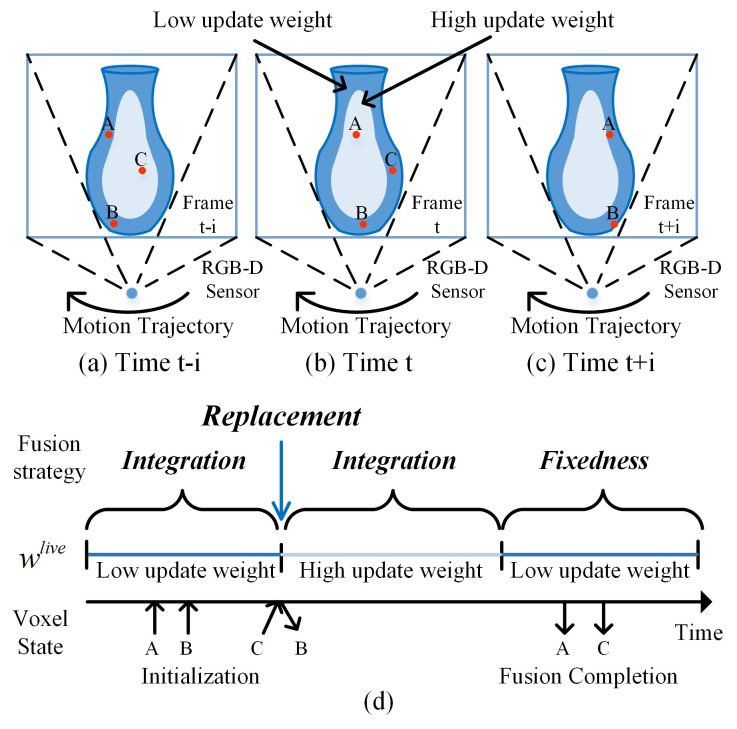
Our texture fusion strategy combines replacement, integration, and fixedness operation based on the updated weight of real-time frames. (**a**–**c**) represent the position of scene voxels “A”, “B”, and “C ”at different times and the estimated weight state of corresponding real-time frames, respectively. (**d**) represents the different state processes that the scene voxels may experience. The arrows represent the beginning and the end of voxel updates.

As shown in Figure 4, where “A”, “B”, and “C” represent three types of voxels that undergo different weight updating processes when the camera view changes with time. The voxel state in this figure represents the updating period from the beginning to the end of voxel “A”, “B”, and “C” over time. The up arrow indicates the start time, and the down arrow indicates the end time. The frame weight $w^{live}$ in the lower part of this figure stands for the confidence weight change of the frame data corresponding to voxel at different times. Figure 4a–c show the position of “A”, “B”, and “C” voxels, and the different confidence weight of the frame data at different times. The light blue represents the high-reliability data area in the frame. On the contrary, the dark blue represents the low-reliability data area in the frame.

Specifically, in this figure, “A” represents voxels that undergo a complete updating period and their confidence weights of frames change from low to high and then to low again with time, which means it will exist in the whole operation of replacement, integration, and fixedness. “B” represents voxels that undergo the updating process with the only low weight of frames, which means that it only uses the integration operation to keep the texture continuity because of a lack of reliable data. “C” represents voxels that undergo the updating process from high to low weight change after the voxels are initialized, which means it will experience integration and fixedness operations.

Since the fusion process of “A” includes “B” and “C”, we take “A” as an example to illustrate our method in detail. For a full updating period of a single voxel, firstly, the texture weight of the voxel is initialized to 0. When the real-time frame’s weight is low, the voxel texture is integrated to keep the continuous, as shown in Figure 4a. When the real-time frame’s weight changes from low to high, the voxel texture is replaced directly by the real-time frame’s texture, and the voxel weight is updated at the same time, this step is performed when voxel “A” is at the boundary of the dark blue and bright blue areas in Figure 4a. When the real-time frame’s weight is maintained at a high level, the texture and weight of the voxels are integrated again. At this time, because the texture has high reliability, we can be assumed that its color difference is very small and there is no blur problem caused by integration operation, as shown in Figure 4b. When the real-time frame’s weight changes from high to low, we fixed the texture and weight of the voxels, to ensure that the high-confidence information is no longer affected by the data stream with low-reliability information, and obtain high-precision reconstruction results, this step as shown in Figure 4c.

The pseudocode of our new texture fusion strategy is detailed in Algorithm 1. Where $w^{live}$ and $w^{pre}$ are texture weights corresponding to voxels of real-time frames and previous frames. $c^{live}$ and $c^{pre}$ are RGB values corresponding to the real-time frames and the voxels of previous frames. $w^{update}$ and $c^{update}$ are texture weights and RGB values updated based on fusion strategy. In this pseudocode, lines 3–4 represent low weight integration operation, lines 6–7 represent fixedness operation, lines 13–14 represent the replacement operation, and lines 16–17 represent high weight integration operation. By computing the confidence of adaptive weighted field estimation with camera motion, we constantly update voxels to preserve the texture structure of the high-confidence region and the continuity of the low-confidence region to ensure that the reconstructed scene has a clearer texture result.

We add the replacement operation mainly considering that the high-weight information in the image domain is very close to the real texture structure, but the texture of the mesh usually contains errors. Instead of using weighted averaging to correct texture errors gradually, it is better to use replacement operation to discard worse date and use more reliable information to obtain higher clarity texture results. At the same time, it can also retain its texture structure. We further introduce fixedness operation to maintain the results of high-weight information fusion, which make it no longer affected by low-weight information containing wrong textures.

We still retain the integration operation mainly considering that for the counterparts between the real-time frame and previous frame fusion result with high-reliability, the probability of mismatching is very low, so we fuse high-quality data to approximate the true value. For the counterparts with low-reliability, the information errors between them are very large, if we replace or fix them into the fusion result, it tends to cause local discontinuity, so we keep the integration operation to smooth the results.
**Algorithm 1** Texture Fusion Strategy**Input:**$w^{live}$, $w^{pre}$,$c^{live}$, $c^{pre}$**Output:**$w^{update}$, $c^{updata}$1:  **if**
$w^{live}$ is low **then**2:  **if**
$w^{pre}$ is low **then**3:   $c^{updata}=\frac{c^{live}\cdot w^{live}+c^{pre}\cdot w^{pre}}{w^{live}+w^{pre}}$
4:   $w^{update}=\frac{w^{live}+w^{pre}}{2}$
5:  **else**6:   $c^{updata}=c^{pre}$
7:   $w^{update}=w^{pre}$
8:  **end if**9:  **end if**10:11: **if**
$w^{live}$ is high **then**12:  **if**
$w^{pre}$ is low **then**13:   $c^{updata}=c^{live}$
14:   $w^{update}=w^{live}$
15:  **else**16:   $c^{updata}=\frac{c^{live}\cdot w^{live}+c^{pre}\cdot w^{pre}}{w^{live}+w^{pre}}$
17:   $w^{update}=\frac{w^{live}+w^{pre}}{2}$
18:  **end if**19: **end if**

By controlling the range of high weight in the adaptive weight field, we can minimize the number of voxels whose reconstruction period is complete in the low update weight stage, such as the voxels undergoing the “B” process. So almost all voxels can experience replacement operations to obtain high-quality information, and finally, the blurring problem can be greatly reduced or even ignored.

## 5. Adaptive Weight Field of RGB-D Image

Our adaptive weight field of RGB-D images follows two basic principles. The first is to effectively model some factors affecting geometric and texture results, such as texture structure, wrong texture, and edge outlier noise. The second is to distinguish high-reliability data by increasing the weight difference based on data reliability assessment. Combined with the above two principles, the adaptive weight field is established.

To satisfy the first principles, we propose a method combining probability and heuristics to model various factors and evaluate the reliability of the data.

To achieve the second principle, we introduce some kernels into the quantization of weight. These kernels will not affect the validity of the reliability estimation results but will increase the distinction between low-reliability data and high-reliability data, thereby reducing the impact of low-reliability data on the subsequent fusion process.

### 5.1. Color Adaptive Weight Field

We build an adaptive weight field of color image mainly to solve the problem of texture blur and wrong texture. For the former, we evaluate the quality of texture features consistency in the image domain to ensure that the mesh maintains a consistent texture structure. For the latter, we mainly suppress the reliability of error-prone areas to avoid the generation of the wrong texture. Besides, we need to effectively adjust the update range of data, so our color weight field can be represented as

(4) $$F^{c}\left(u,v\right)=W_{photo}^{c}\cdot W_{occl}^{c}\cdot W_{view}^{c}$$

where $W_{photo}^{c}$ is photometric consistency weight field used to evaluate the reliability of local texture features, $W_{occl}^{c}$ is edge error weight field used to mark areas prone to errors and suppress its effects, $W_{view}^{c}$ is color viewpoint dependent weight field used to control the range of texture updates.

#### 5.1.1. Photometric Consistency Weight Field

The current real-time reconstruction method usually does not consider how to keep the texture structure characteristics of the mesh, but it’s quite important to avoid texture blur. So we build the photometric consistency weight field to preserve the mesh texture structure in the fusion process. The main method is to ensure that the texture provided to the global model for updating should have high structure consistency and data reliability. The consistency and reliability in the image domain can be obtained by evaluating the texture feature difference between the real-time frame and the previous frame. It should be emphasized that texture features are different from texture colors. The former describes the texture structure of local neighborhood pixels, while the latter only describes the color of a single pixel.

If the difference of texture feature between frames in some regions is very small, such texture is assigned with high reliability and consistency weight in texture fusion. On the contrary, if the difference between frames in some regions is large, the related texture data should be excluded in the texture fusion. The poor consistency of texture features may occur on surfaces with large geometric gradients. Based on the above idea, we design a photometric consistency weight field to evaluate the consistency of the local texture feature of the real-time frame.

Although many features can be used to describe the local texture structure, such as SIFT, SURF, and ORB, etc, these features are generally sparse and can’t effectively evaluate the dense local texture features through the whole image. Therefore, we propose a new texture feature to describe the local texture structure.

With the inspiration of the Laplacian coordinate that can well describe the local geometric feature, we introduce the Laplacian texture coordinates to describe the local texture features in the image domain. Our Laplacian texture coordinates are defined by photometric values, which can ensure the invariance of texture rotation and translation, and facilitate the calculation of the weight field. To obtain more accurate texture features, different from directly using Euclidean distance to evaluate the difference between adjacent points in geometry, we consider that the change of color in RGB space is usually anisotropic, so we use Mahalanobis distance to calculate the difference between textures. Our Laplacian texture coordinates can be written as

(5) $$\delta_{i}=c_{i}-\frac{\rho_{ij}}{\sum_{j=0}^{m}\rho_{ij}}c_{j}$$

where $\delta_{i}$ is the Laplacian texture coordinate on pixel *i*, $c_{i}$ and $c_{j}$ are the photometric values corresponding to pixel i and its neighborhood pixel j. m represents the number of neighbor pixels at pixel i, which can be 4 or 8. Generally, an 8-neighborhood calculation can be used for rich texture, and 4-neighborhood can be selected for faster calculation speed. $\rho_{ij}$ describes the color difference between pixel i and pixel j, which can be calculated by Mahalanobis distance and is written as

(6) $$\rho_{ij}=\sqrt{{\left(r_{i}-r_{j}\right)}^{Τ}\sum_{r_{i}}^{-1}\left(r_{i}-r_{j}\right)}$$

where $r_{i}$ and $r_{j}$ are RGB color at pixel i and pixel j. $\sum_{r_{i}}^{-1}$ is the inverse of the covariance matrix about $r_{i}$.

To evaluate the consistency of texture features, we first calculate the dense texture feature map for the real-time frame and the previous frame respectively. Then we calculate the texture feature difference between them based on the estimated camera pose and projection model and establish the photometric consistency weight field. This weight field can be calculated by

(7) $$W_{photo}^{c}=\{\begin{array}{cc} 1.0 & \left\|\delta^{l}-\delta^{p}\right\|<=t_{photo}\\ 1.0/e^{\lambda \left\|\delta^{l}-\delta^{p}\right\|} & other \end{array}$$

where $\delta^{l}$ and $\delta^{p}$ represent the corresponding texture features between the real-time frame and the previous frame. $t_{photo}$ is a constant threshold value used to control the reliability calculation of texture features and is set to 0.1. λ is another constant used to control the decay rate of reliability and is set to 1.

#### 5.1.2. Edge Error Weight Field

**Figure 5:**
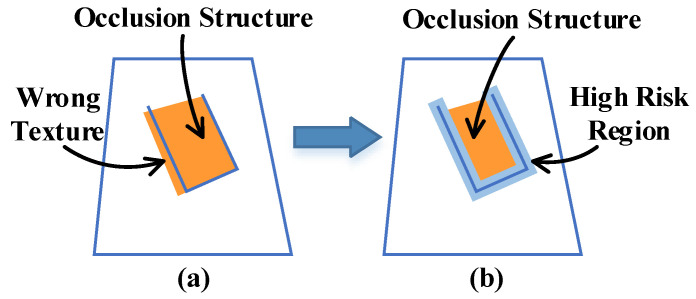
Description of edge error weight field modeling. (**a**) shows the problem of the wrong texture in the edge area of the scene, (**b**) shows that the high-risk area can be marked and the data reliability of these areas can be adjusted to avoid the wrong texture.

Texture errors mainly occur in the edge area of the scene, especially when there is an occlusion structure. As shown in Figure 5a, this is mainly because the texture at the edge usually changes dramatically, and the texture on both sides of the edge is very easy to mix and produce the wrong texture results. When there are large errors in camera pose evaluation, this problem will become very obvious. However, in addition to using off-line optimization to correct these wrong textures, the current method does not have a good strategy to avoid such a problem. For this reason, we establish an edge error weight field to suppress the reliability of error-prone areas.

The core idea is to select the edge of the scene, evaluate its influence range, and then set its weight to 0. Through these operations, the edge area can no longer affect the high-quality texture that has been fused previously, thus avoiding the occurrence of the wrong texture. Because the scene information collected by RGB-D is highly redundant, the texture of these regions can be obtained by fusing the high-reliability data from other viewpoints without being affected by the edge.

We first extract all edges of the scene according to the angle between the viewpoint and the normal vector of the mesh surface. To determine its influence range, we perform a morphological expansion operation on these edges to obtain boundary areas, which is the high-risk region be shown in Figure 5b, and we need to ensure that these areas are not affected by the edge. Finally, the edge error weight field is calculated.

(8) $$W_{occl}^{c}=1.0-E\left(M\left(1.0-a\cos\left(\frac{l_{view}\cdot n}{\left|l_{view}\right|\cdot \left|n\right|}\right)\frac{180}{t_{occl}\cdot \pi }\right)\right)$$

where $t_{occl}$ is the truncation angle constant is used to extract edges, usually set 70°. E(⋅) is the expansion operation for the image to cover the influence range. M(⋅) is a distinguishing function to mark the high-risk region of the edge and can be written as

(9) $$M\left(x\right)=\{\begin{array}{cc} 1.0 & x>0.9\\ 0 & else \end{array}$$

According to the definition of M(⋅), if a pixel is in the influence range, its weight is set to 0, otherwise, it is set to 1. A weight of 0 means that the texture information in the influence range has a high risk of error, so we do not use its data to update the mesh texture.

#### 5.1.3. Viewpoint Dependence Weight Field

Viewpoint-dependent uncertainty is a widely accepted and effective description of noise distribution in the depth map. However, in the color image, we use viewpoint dependence mainly to evaluate the texture quality of the surface and control the range of texture update. These two points can be realized by adjusting the model parameters by establishing the view-dependent weight field.

We first calculate the viewpoint dependent uncertainty $u^{view}$.

(10) $$u^{view}=\min(a\cos\left(\frac{l_{view}\cdot n}{\left|l_{view}\right|\left|n\right|}\right)\cdot \frac{180}{t_{view}\cdot \pi },1.0)$$

where $l_{view}$ is the viewing direction, n is the normal computed from the input depth map, $t_{view}^{d}$ is an angle constant about normalization.

Then we calculate the color view-dependent weight field by

(11) $$W_{view}^{c}=\varphi_{view}^{c}\left(1.0-u^{view}\right)$$

where $\varphi_{view}^{c}\left(\cdot \right)$ is truncation kernel functions to increase the difference between reliability and non-reliability and defined as

(12) $$\varphi_{view}^{c}\left(x\right)=\{\begin{array}{cc} x & x>t_{threshold}^{c}\\ 0.1\cdot x & x\leq t_{threshold}^{c} \end{array}$$

where $t_{threshold}^{c}$ is a truncation constant as high confidence.

We use low coefficients to reduce the weight instead of setting it to zero directly, mainly to keep the low-weight data continuous when texture fusion.

Here we use this kernel function mainly considering that the texture fusion process is more sensitive to the difference between high-quality data and low-quality data of RGB image, and the greater difference is conducive to better texture reconstruction effect.

When the texture data is on a relatively smooth surface and the angle between the normal vector of the surface and the opposite direction of the viewpoint is small, we can think that the texture is better and its reliability is higher. So, based on the above model, we can find that by adjusting parameter $t_{view}$, we can effectively control the reliability of the texture data and its range. The larger $t_{view}$ setting, the higher the reliability, but its range will be reduced. Conversely, the smaller $t_{view}$ setting, the lower the reliability, but its range will be increased.

### 5.2. Depth Adaptive Weight Field

For the geometric fusion error, we mainly consider the influence of noise in the foreground edge area of the depth map. Because of these areas usually contain a large number of outliers and large-scale noise caused by environmental illumination, measurement methods, and other factors, they cannot be accurately described by the existing models. More importantly, this noise will destroy the reconstructed surface, reduce the surface quality, and slow down the convergence speed. Therefore, it is necessary to model to suppress the noise influence in the edge area.

#### Edge Noise Weight Field

The current methods usually use bilateral filtering to deal with the noise in the foreground edge region, but this method cannot effectively remove large-scale outliers, which often lead to obvious geometric fusion errors. Therefore, we establish a heuristic and probability edge noise model, which can effectively remove outliers and evaluate the noise distribution in these areas.

The core idea is to remove these outliers by heuristic method firstly, and then use the probability method to evaluate the reliability of the information at the edge.

We use depth truncation and region growth to obtain the foreground of the target scene in the depth image. Then heuristically, we divide the foreground pixels into three categories: outer point, edge region point, and interior point. Outer points refer to the pixels in an image that are not related to the target scene. Edge region points mainly refer to points extending along the edge of the scene to the interior, and they form a strip area. Their noise distribution can be approximated by a Gaussian probability model. The interior points refer to the points in the interesting region.

We calculate the Manhattan distance $d_{edge}$ from each edge pixel to the nearest interior point. Finally, we use the following formula to calculate the edge weight field of depth image

(13) $$W_{edge}^{d}=Gauss\left(\rho_{edge}\right)$$

where Gauss(⋅) is a Gaussian blur operation to fit the noise which conforms to the Gaussian distribution at the edge, $\rho_{edge}$ is the initial edge weight field which has almost eliminated the influence of outliers in the scene by a heuristic method and is defined as

(14) $$\rho_{edge}=\{\begin{array}{cc} 0 & outer\\ d_{edge}/t_{edge} & edge\\ 1 & inner \end{array}$$

where $t_{edge}$ is the influence width of the scene edge region. It means the strip region extending $t_{edge}$ pixels to the edge of the foreground.

## 6. Refined Camera Pose Evaluation

Considering that our reconstruction results have high-quality texture, we propose a two-step refined camera pose estimation strategy combining our adaptive weight field, fused geometry, and texture information. Firstly, we use a frame to frame ICP to obtain the initial relative pose between the real-time frame and the previous frame. Secondly, we refine the projection matrix T by registering the real-time frame to the previous reconstruction of global mesh. This registration is achieved by minimizing an energy function which consists of two terms: the geometric vertex error term and the photometric consistency error term and is written as

(15) $$E=\sum_{(p_{d},p_{m})\in P}w_{d}{\left(p_{d}-T^{-1}p_{m}\right)}^{2}+{\sum_{(c_{d},c_{m})\in C}w_{t}(c_{d}-c_{m})}^{2}$$

where $p_{m}$ is the vertex of the model, $p_{d}$ is the projection vertex of real-time depth frame D corresponding to $p_{m}$ and satisfies $p_{d}=\pi^{-1}(D(\pi (T^{-1}p_{m})))$. P describes this projection correspondence. $c_{m}$ is the photometric value of the mesh vertex $p_{m}$, $c_{d}$ is the photometric value of the corresponding mesh vertex $p_{m}$ projected on the RGB image I and satisfies $c_{d}=I(\pi (T^{-1}p_{m}))$.

Different from the general frame to model ICP methods using 1.0 as the weight of error term and does not consider the texture information, we use the dynamically changing weight $w_{d}$ and $w_{t}$ and introduce the photometric consistency as another effective constraint. $w_{d}$ and $w_{t}$ are fused geometry and texture weights corresponding to model vertices calculated from our adaptive weight field and are varies in the different places. According to this changing weight mechanism, our pose refinement method can use more reliable geometric and texture information to adjust the pose and improve the accuracy of the reconstruction result.

The energy function in the form of least square can be effectively solved by a GPU style Gauss–Newton iteration. The refined pose results are stored and used for the updating of the mesh.

We use a two-step pose estimation mainly to improve the robustness of frame to model registration because with the increase of time, the relative pose between the real-time frame and global model will increase, and the estimated pose error will be larger, which will lead to easy tracking failure, especially for incomplete surfaces. So we use the frame to frame estimation results to provide a better initial pose and further improve the robustness of frame to model pose estimation process.

## 7. Results

We have designed three experiments to verify the effectiveness of the new fusion strategy, adaptive weight field, and two-step refined pose estimation, respectively. Firstly, we compared our method with the current mainstream online and offline methods in qualitative and quantitative ways to illustrate the effectiveness of our method. Secondly, we have illustrated the effectiveness of the adaptive weight field in improving texture quality by establishing a real reference experimental platform [36]. Finally, we use real scene datasets to compare the cumulative pose errors from four different pose estimation strategies to shows the effectiveness of our two-step pose estimation method.

In the first experiment, we embedded the new volumetric fusion method into the framework of KinectFusion, and compared the public RGB-D dataset [20] and generated datasets with the online KinectFusion [6] and the off-line Intrinsic3d [5] to evaluate the effectiveness of our method qualitatively and quantitatively. The RGB-D data are obtained by using a ToF sensor. Our assessment is implemented on a platform with an Intel Core i7-8700K CPU with 3.70GHz and 16GB RAM.

We set $t_{edge}=15$ in (14), $t_{threshold}^{c}=0.7$ in (12) and $t_{view}=70{}^{\circ}$ in (10) according to experience. The experimental results show that these parameter settings can robustly reconstruct a variety of scenes, including relief or complex rotator, with good reconstruction accuracy. Firstly, we use the literature method [30] to select keyframes from the dataset to add the noise to enhance the effect comparison, and then select 1.0 mm voxel size for RGB-D reconstruction.

### 7.1. Qualitative Analysis

The public RGB-D dataset we use is shown in Table 1. In the KinectFusion, we use a viewpoint dependence weight calculation method [5] defined as

(16) $$w_{KF}=\frac{m}{3}\phi \left(1.0-a\cos\left(\frac{l_{view}\cdot n}{\left|l_{view}\right|\left|n\right|}\right)\right)+\frac{m}{3}\phi \left(2\frac{\left|d\right|}{d_{tr}}\right)+m\cdot norm\left(p_{z}\right)$$

where m is a fusion constant equal to 10, d is the truncated signed distance function (TSDF) value corresponding to voxels, $d_{tr}$ is the truncation distance of voxels equal to 5.0 mm, $p_{z}$ is the depth of a pixel calculated in camera coordinates. norm(⋅) is a function to normalizing the depth value $p_{z}$. ϕ(⋅) is a robust kernel function and defined as

(17) $$\phi \left(x\right)=\frac{1.0}{{\left(1.0+2\cdot x\right)}^{3}}$$

This weight calculation method effectively considers the uncertainty of viewpoint angle, distance, and TSDF and is the representative fusion weight calculation of the classical voxel method. However, this weight calculation method mainly aims at depth map, and does not deal with the possible texture blur and wrong texture problems of the color image, and also does not consider the outlier noise at the edge of the depth map. This means that they may only get poor texture results.

**Figure 6:**
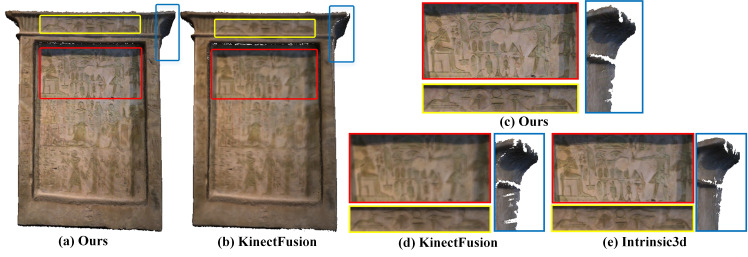
The reconstruction results of the Hieroglyphics dataset. Our results (**a**,**c**) are better in geometry and texture than the classical weighted average fusion method [6] (**b**,**d**), and can reach the level of offline optimization of intrinsic3d reconstruction framework [5] (**e**).

**Figure 7:**
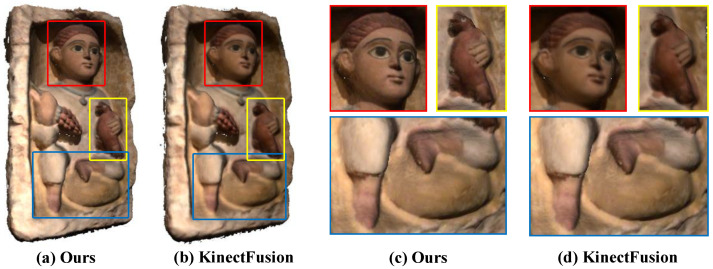
The reconstruction results of the Tomb Statuary dataset. Comparing with the classical weighted average fusion method [6] (**b,d**), our results (**a,c**) can achieve better texture clarity and color fidelity.

**Figure 8:**
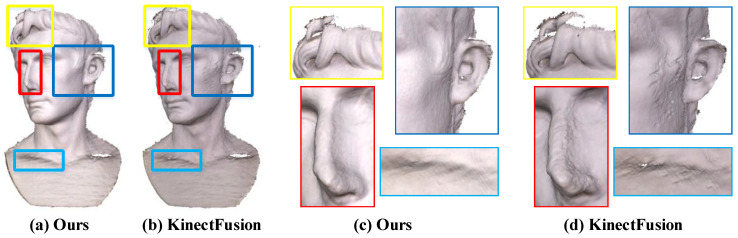
The reconstruction results of the August dataset. Comparing with the classical weighted average fusion method [6] (**b,d**), our results (**a,c**) can deal with the details of geometry.

Experimental comparisons are given in Figure 1 and Figure 6, Figure 7 and Figure 8.

By comparing Figure 1, Figure 6, and Figure 7, it can be found that our method can effectively keep the local texture structure information of the reconstructed mesh, and solve the problem of texture blur robustly. Higher clarity texture effects can be obtained compared to the offline methods based on joint optimization, such as Intrinsic3d [5]. On the one hand, this excellent texture clarity comes from the adaptive weight field to ensure that the texture data used in the texture fusion process has high reliability and features consistency. On the other hand, it comes from the new texture fusion strategy that combines replacement, integration, and fixedness operation can effectively preserve texture structure features in the texture update process. The effectiveness of clarity and fidelity are illustrated in the Supplementary Materials in detail.

By comparing the results of geometric reconstruction in Figure 8, it can be found that our method can better suppress the noise of the depth map, and obtain a more complete and smooth surface than the online KinectFusion [6] method. This is mainly because we consider edge noise to effectively reduce the influence of edge outliers on the surface fusion results, thus accelerating the surface convergence process.

### 7.2. Quantitative Analysis

To show that our method can effectively suppress the influence of noise, we use the Sokrates model [20] to generate true RGB-D data and true camera pose, and then add 0.1 mm, 0.5 mm, and 1.0 mm Gauss noise to RGB-D image respectively, and add slight perturbations to camera pose to obtain the synthetic RGB-D data. With these data, we quantitatively compare the geometric accuracy and texture accuracy of the reconstruction results between KinectFusion and our approach by using both 1.0 mm-sized voxels and 2.0 mm-sized voxels.

**Figure 9:**
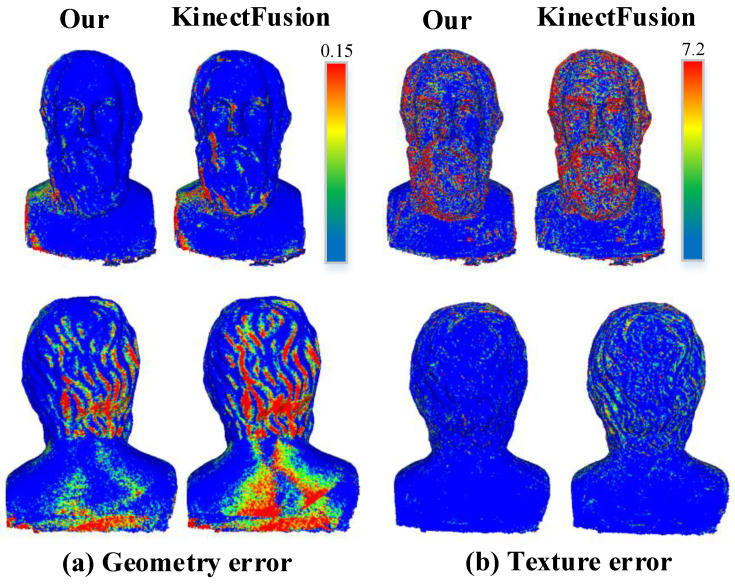
Quantitative analysis of geometry (**a**) and texture (**b**) based on our method and traditional weighted average fusion method [6].

We use the Square Sum Error (SSE) and Mean Square Error (MSE) of the geometric position and texture color between the reconstruction model and the standard model to evaluate the accuracy of the reconstruction results. The results are shown in Figure 9, and Table 2 and Table 3.

As shown in Figure 9, we can find that our method can significantly reduce geometric and texture errors compared with the KinectFusion method, especially in the region with large surface fluctuations. The obvious improvement mainly benefits from the depth edge noise weight field avoids the interference of outlier noise on the surface, and our two-step refined pose estimation results avoid the surface discontinuity or the jittery effect caused by pose error.

As far as the accuracy of the geometric reconstruction is concerned, as shown in Table 2, with the increase of noise level, our method can improve the accuracy by 15–25% comparing with the KinectFusion fusion method under the condition of 1.0 mm voxel size and 22% under the condition of 2.0 mm voxel size.

From the texture accuracy comparison, as shown in Table 3, it can be drawn that our method can steadily improve the accuracy by about 20% under the condition of 1.0 mm sized voxel or 2.0 mm sized voxel to the classical fusion method under different noise levels.

### 7.3. Adaptive Weight Field

**Figure 10:**
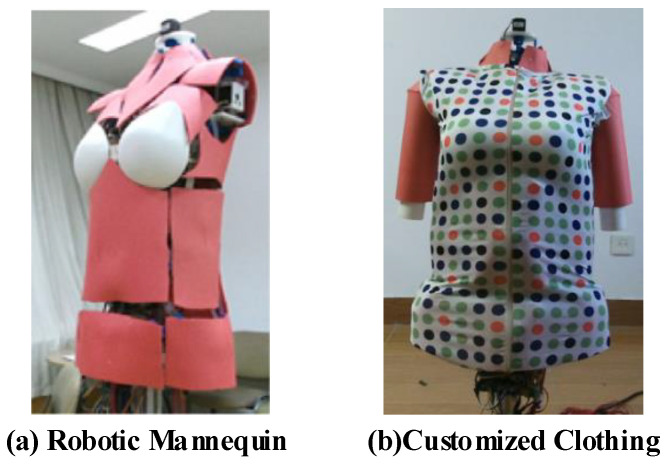
A real reference experimental platform. (**a**) we use a robotic mannequin developed in our lab [36]; (**b**) the clothing on the robotic mannequin will be reconstructed to test the adaptive weight field.

In the second experiment, we use a robotic mannequin [36] and custom-made clothing to build a reference experimental platform for real scene reconstruction effect comparison to illustrate the effectiveness of our adaptive weight field. This experimental platform is shown in Figure 10.

The custom-made clothing contains many different colored and disc-shaped textures, as shown in Figure 10b, which are used to effectively evaluate the structural consistency of the reconstruction results. We put the customized clothing on the robotic mannequin, keep a distance of about 1.0 m, and hold the Kinect V2 depth camera to rotate around the robot for one circle to obtain the corresponding RGB-D dataset. To show the improvement effect of the adaptive weight field more clearly, we only use a frame to frame ICP to estimate the camera’s pose, which means that there will be large error factors in geometry and texture updating. Such errors can be greatly reduced by adopting the adaptive weight field as follows.

**Figure 11:**
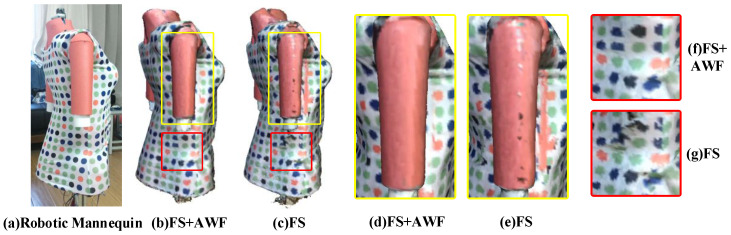
The validation of the color adaptive weight field, (**a**) is the used robotic mannequin and customized clothing; (**b**,**d**,**f**) are the results obtained by using the new fusion strategy (FS) and the adaptive weight field (AWF); (**c**,**e**,**g**) are the results obtained only by using the new fusion strategy (FS).

**Figure 12:**
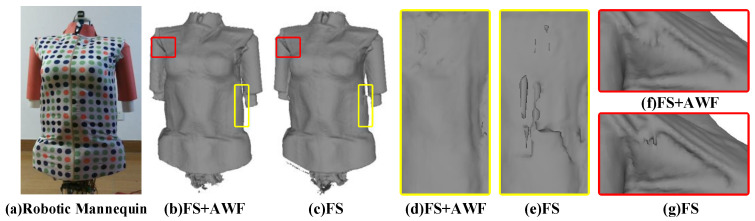
The validation of the depth adaptive weight field, (**a**) is the used robotic mannequin and customized clothing; (**b**,**d**,**f**) are the results obtained by using the new fusion strategy (FS) and the adaptive weight field (AWF); (**c**,**e**,**g**) are the results obtained only by using the new fusion strategy (FS).

We design two kinds of reconstruction frameworks, one of which only uses the new fusion strategy (FS), the other uses the combination of adaptive weight field (AWF) and new fusion strategy (FS). During the experiment, we used the same parameters as the first experiment but set the resolution of the voxel grid to 4 mm. We study the influence of color adaptive weight field on texture reconstruction and depth adaptive weight field on geometry reconstruction. The results are shown in Figure 11 and Figure 12.

Figure 11 illustrates the effectiveness of the color adaptive weight field. By comparing Figure 11e,d, it can be found that by using the edge error weight field, the problem of wrongly mixing the texture from two sides of the edge can be avoided. The comparison between Figure 11f,h shows that the proposed photometric consistency weight field can effectively preserve the local texture structure features, making the reconstructed texture results accurate. Figure 11b,c prove that our color adaptive weight field can significantly improve the quality of texture results and obtain more accurate and realistic texture effects.

Figure 12 is a comparative experiment to illustrate the improvement of depth adaptive weight field on geometric surface reconstruction. By comparing the reconstructed surface quality obtained by different methods shown in Figure 12d–g, it can be demonstrated that using the edge noise weight field can successfully suppress the interference of edge outliers on the reconstruction process, accelerate the convergence speed of the surface. A smoother and more accurate geometric result can be obtained.

### 7.4. Two-Step Refined Pose Estimation

To illustrate the effectiveness of our two-step pose estimation method, we compared the accumulated pose error from the frame to frame ICP method (FF-ICP), the frame to model ICP method (FM-ICP), our two-step pose estimation method without adaptive weight (TSN-ICP) and two-step pose estimation method with adaptive weight (TSW-ICP) using the real scene RGB-D dataset collected based on our experimental platform. The difference between TSN-ICP and TSW-ICP is that the former uses the same weight for each data item, while the latter uses different weights for each data item, and these weights are based on the adaptive weight field.

**Figure 13:**
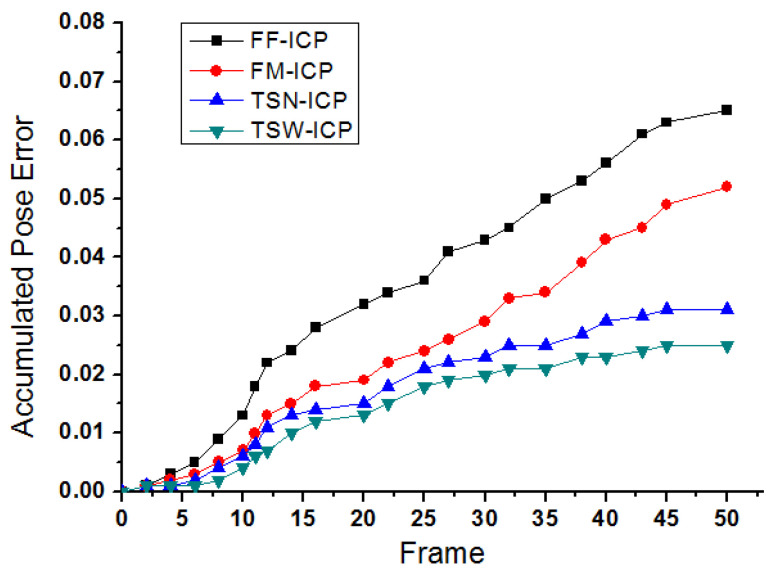
The result of accumulated pose error from frame to frame ICP method (FF-ICP), the frame to model ICP method (FM-ICP), two-step pose estimation method without adaptive weight (TSN-ICP) and two-step pose estimation method with adaptive weight (TSW-ICP). The results show that TSW-ICP can effectively improve the accuracy of pose estimation.

Based on our experimental platform, using the same scene and data acquisition method as the second experiment, we collected an RGB-D dataset containing 479 frames and uniformly sampled and selected 50 frames as keyframes to test the pose accumulative error of different pose estimation methods. We use the off-line global optimization method to get the pose of our dataset to approximate the true value to calculate the pose error, such as the method proposed by Zhou [12]. Therefore, we carried out comparative experiments for four different ICP methods, and the results are shown in Figure 13.

By comparing the changing trend of accumulated pose error in Figure 13, it can be found that there will be obvious drift problems in the frame to frame ICP method (FF-ICP) because the pose error between each frame is accumulating. For the frame to model ICP method (FM-ICP), the estimated error is small at the beginning, but with the increase of the relative pose between the real-time frame and the model with time, the convergence of pose estimation becomes more difficult, which leads to the increasing of the estimated error, and it is prone to track failure. Compared with the FF-ICP method and the FM-ICP method, our two-step pose estimation method can significantly reduce the accumulated pose error, and the TSW-ICP methods with the adaptive weight field can further improve the pose accuracy.

Although the calculated cost of two-step optimization is slightly higher than that of one-step FF-ICP and FM-ICP, the calculated cost proportion of camera pose estimation is relatively small in the whole reconstruction process. Therefore, to significantly improve the accuracy of reconstructed results, this cost is acceptable compared with the offline pose optimization method.

### 7.5. Limitations

Although our new texture fusion method and adaptive weight field can effectively preserve texture structure, avoid the wrong texture, and suppress outlier noise, there are still some problems that limit the application of our method. The lighting illumination factor is not considered in the adaptive weighting field. Therefore, currently, our method cannot robustly deal with scenes with obvious illumination changes. Considering the lighting illumination usually requires physical-based rendering, it requires a large computational cost and makes it hard to run in real-time. In the future, the simplified spherical harmonic illumination model may be used to obtain some degree of illumination evaluation and effect improvement. For a similar reason, our method is mainly applied to reconstruct the scene with Lambert material, which may cause a large error in reconstructing a scene with specular reflective materials. In our method, texture discontinuity may occur when there is a large error in the camera pose evaluation. However, our TSW-ICP method can obtain good enough camera poses without obvious discontinuity.

## 8. Conclusions

We have proposed an adaptive weight field in image space to effectively evaluate the reliability of RGB-D data to preserve texture structure, avoid wrong texture, and suppress outlier noise. Based on our adaptive weight field, a new texture fusion strategy using the combination of replacement, integration, and fixedness has been designed. Because this method can effectively preserve the local texture structure information of the global mesh, it can achieve an excellent texture clarity compared with the online KinectFusion and offline method based on joint optimization, such as Intrinsic3d. In addition, a two-step pose estimation method considering geometry and texture information is also proposed to refine the estimated pose, which can further improve the quality of reconstruction results and avoid the discontinuity of texture.

More importantly, our method can be easily embedded into various online and offline reconstruction frameworks as a basic module, and can significantly improve the reconstruction quality, especially for texture.

Further work is required to explore an efficient environmental illumination assessment model, which can robustly solve the impact of illumination on our algorithm and reconstruct a wider range of scenes and may be achieved by introducing a simplified spherical harmonic illumination model. On the other hand, we plan to extend the current method to the reconstruction of non-rigid scenes.

## Figures and Tables

**Table 1:** The RGB-D dataset for evaluation.

Dataset	Resolution		Frames	Keyframes
	Depth	Color		
Gate [5]	640 × 480	1296 × 968	1213	61
Hieroglyphics [5]	640 × 480	1296 × 968	919	46
Tomb Statuary [5]	640 × 480	1296 × 968	523	27
Lion [5]	640 × 480	1296 × 968	514	26
Bricks [5]	640 × 480	1296 × 968	773	39
Lucy [20]	640 × 480	640 × 480	99	99
Augustus [20]	640 × 480	1280 × 1024	73	73
Socrates [20]	1139 × 1709	1139 × 1709	33	33

**Table 2:** Quantitative analysis results of geometric reconstruction accuracy at different noise levels.

Experimental Condition		Our_SSE	KF_SSE	Our_MSE (10^−3^)	KF_MSE (10^−3^)
Grid Resolution	Gauss Noise				
1 mm	No	**52.676**	58.217	**0.0088**	0.0098
	0.1 mm	**56.331**	63.176	**0.0094**	0.0106
	0.5 mm	**104.676**	175.080	**0.0175**	0.0293
	1.0 mm	**636.720**	845.570	**0.1067**	0.1417
2 mm	No	**15.362**	19.894	**0.0052**	0.0068
	0.1 mm	**16.128**	20.761	**0.0055**	0.0071
	0.5 mm	**34.371**	44.485	**0.0117**	0.0151
	1.0 mm	**104.083**	132.770	**0.0354**	0.0452

**Table 3:** Quantitative analysis results of texture reconstruction accuracy at different noise levels.

Experimental Condition		Our_SSE	KF_SSE	Our_MSE (10^−3^)	KF_MSE (10^−3^)
Grid Resolution	Gauss Noise				
1 mm	No	**8510**	13087	**1.4258**	2.1927
	0.1 mm	**9113**	13849	**1.5269**	2.3204
	0.5 mm	**14998**	19593	**2.5129**	3.2828
	1.0 mm	**25687**	29272	**4.3038**	4.9045
2 mm	No	**2692**	4076	**0.9165**	1.3877
	0.1 mm	**2777**	4196	**0.9455**	1.4286
	0.5 mm	**3855**	5281	**1.3125**	1.7980
	1.0 mm	**5566**	6747	**1.8950**	2.2971

## Supplementary Materials

The following are available online at https://www.mdpi.com/1424-8220/20/15/4330/s1.
