# Supplementary material for: A New Volumetric Fusion Strategy with Adaptive Weight Field for RGB-D Reconstruction
Source: Sensors (Basel). 2020 Aug 3;20(15):4330. doi: 10.3390/s20154330 (PMC7436277; doi:10.3390/s20154330)
Supplement: Supplementary file 1 [file sensors-20-04330-s001.zip › Supplementary Materials-A New Volumetric Fusion Strategy with Adaptive Weight Field for RGB-D Reconstruction.pdf]

sensors

Supplementary Materials

A New Volumetric Fusion Strategy with Adaptive Weight Field for RGB-D Reconstruction

Xinqi Liu [1], Jituo Li [1,*] and Guodong Lu [1]

[1] Zhejiang University, Hangzhou 310027, China; liuxinqi@zju.edu.cn (L.X.); jituo_li@zju.edu.cn (L.J.); lugd@ zju.edu.cn(L.G.)
* Correspondence: jituo_li@zju.edu.cn

Received: date; Accepted: date; Published: date

1. Texture Clarity

We use bricks dataset[2] to further illustrate the improvement of texture clarity by our method. We compare the texture reconstruction effect of online KinectFuison[1], offline Infinsic3D[2] and our method, as shown in Figure 1.

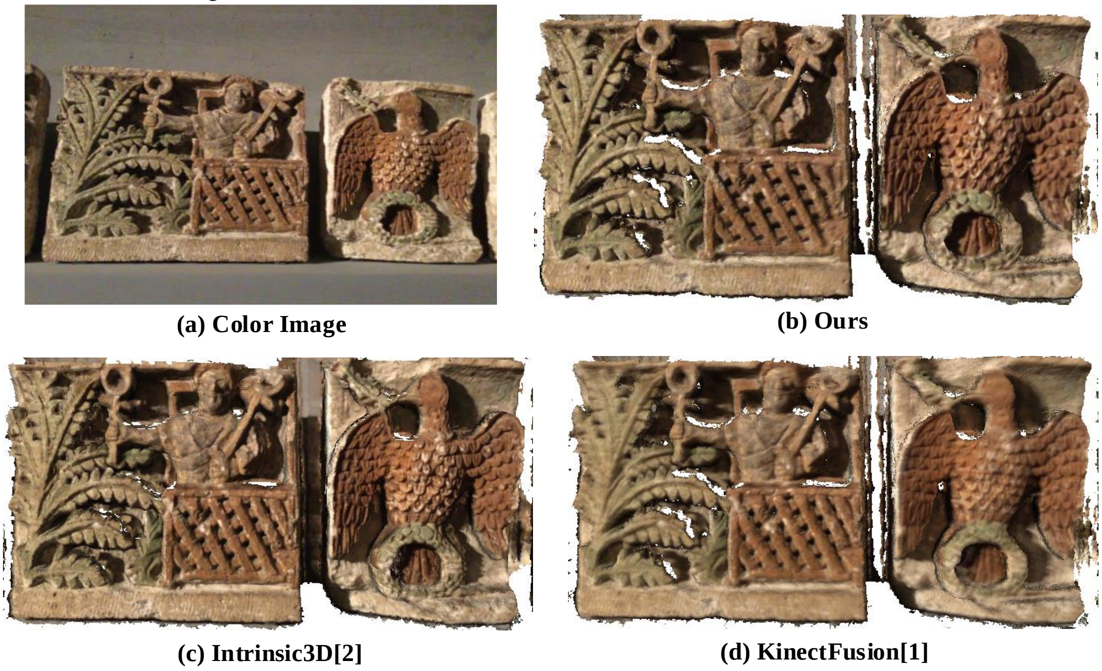

Figure 1. Texture reconstruction results of the Bricks dataset. (a) is the color image of this scene. (b) is the result of our method (a) and it can achieve better texture clarity than the KinectFusion[1] (d) and the off-line intrinsic3D[2] (c) in the place with rich texture structure.

Through comparison with Figure 1, we can find that KinectFusion is very easy to generate blur result on the texture rich geometric surface due to it based on weighted average, while our method can effectively preserve the local texture structure of mesh because we establish an adaptive weight field and use a new texture fusion strategy, so the high-quality texture clarity can be achieved and no less than the offline method.

2. Texture Fidelity

We use Lion dataset[2] and Lucy dataset[3] to show that our method can obtain high fidelity reconstruction results. Because of our adaptive weight field and new texture fusion strategy combines the replacement, the integration and fixedness can well preserve the local texture structure

information of mesh, so we can well present the high fidelity almost similar to the image texture. The texture results are shown in Figure 2 and Figure 3.

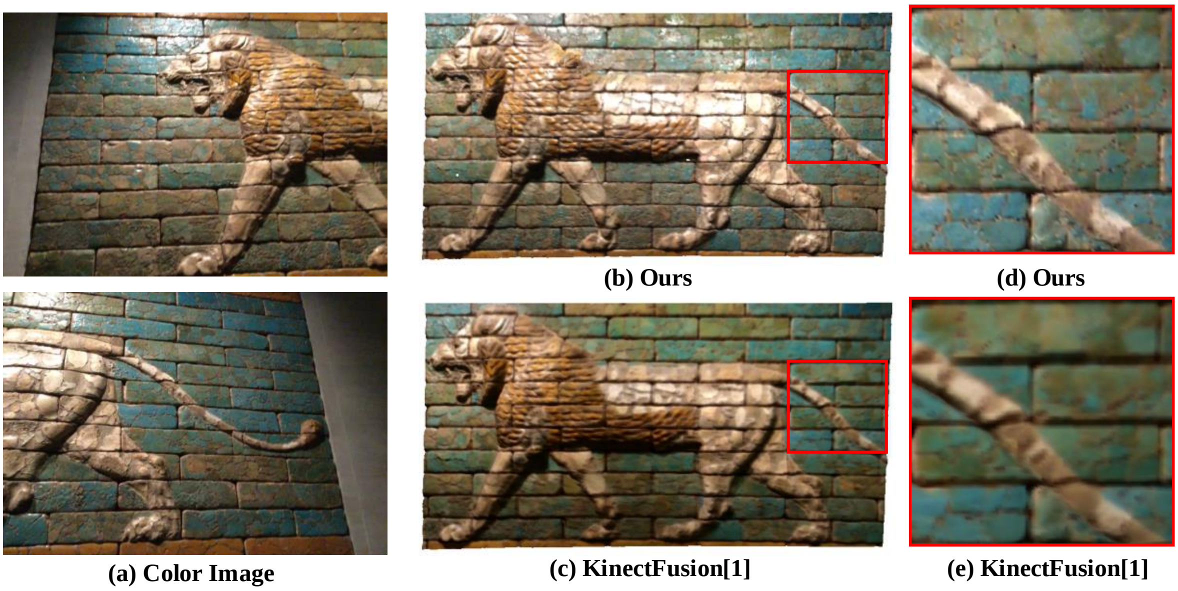

Figure 2. Texture reconstruction results of the Lion dataset. (a) is the color image of this scene. (b) and (d) are the result of our method and it can achieve better texture fidelity and clarity than the KinectFusion (c) and (e).

The results in Figure 2 show that KinectFusion not only loses the details of the surface due to blur, but also produces a certain color bias, which generate a deeper color effect. On the contrary, our method can achieve a higher fidelity, which can be seen by comparing color images.

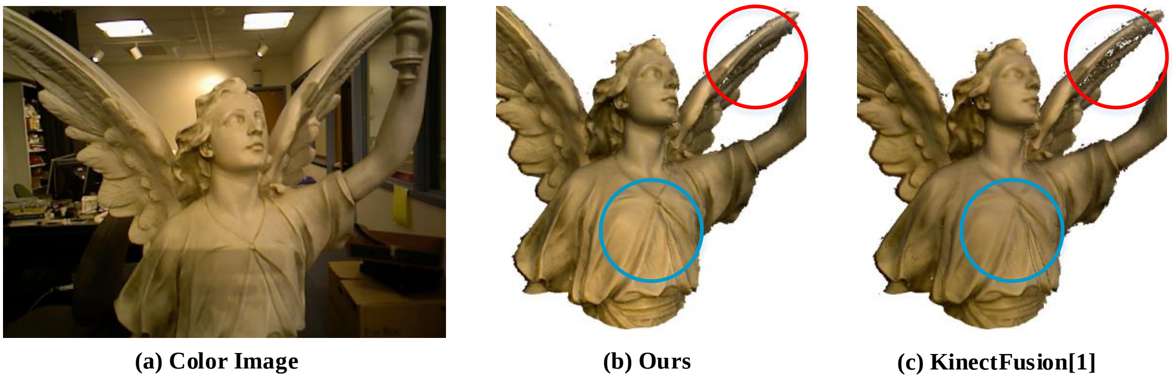

Figure 3. Texture reconstruction results of the Lucy dataset. (a) is the color image of this scene. (b) is the result of our method (a) and it can achieve better texture fidelity and complete than the KinectFusion[1] (c).

By comparison with Figure 3, we can find that our method can obtain a more complete geometry, as shown in the red circle in this figure, and a more accuracy and real texture effect, as shown in the blue circle in the figure, compared with the result of KinectFusion[1].

References

1. Izadi, S.; Kim, D.; Hilliges, O.; Molyneaux, D.; Newcombe, R.; Kohli, P.; Shotton, J.; Hodges, S.; Freeman, D.; Davison, A. KinectFusion: Real-time 3D reconstruction and interaction using a moving depth camera. In Proceedings of the 24th Annual ACM Symposium on User Interface Software and Technology,Santa Barbara, CA, USA , 16–19 October 2011; pp. 559–568.
2. Maier, R.; Kim, K.; Cremers, D.; Kautz, J.; Nießner M. Intrinsic3D: High-Quality 3D Reconstruction by Joint Appearance and Geometry Optimization with Spatially-Varying Lighting. In Proc. IEEE Int. Conf. Comput. Vis. 2017, pp. 3133-3141.

3. Zollhöfer, M.; Dai, A.; Innmann, M.; Wu, C.; Stamminger, M.; Theobalt, C. Shading-based refinement on volumetric signed distance functions. ACM Trans. Graph. 2015, 34, 96.

© 2020 by the authors. Submitted for possible open access publication under the terms and conditions of the Creative Commons Attribution (CC BY) license (http://creativecommons.org/licenses/by/4.0/).
